# Microfluidic High-Q Circular Substrate-Integrated Waveguide (SIW) Cavity for Radio Frequency (RF) Chemical Liquid Sensing

**DOI:** 10.3390/s18010143

**Published:** 2018-01-06

**Authors:** Muhammad Usman Memon, Sungjoon Lim

**Affiliations:** School of Electrical and Electronics Engineering, College of Engineering, Chung-Ang University, 84-Heukseok-ro, Dongjak-gu, Seoul 156-756, Korea; musmanm@outlook.com

**Keywords:** chemical sensor, SIW, circular cavity, RF sensor, wireless sensor, ethanol

## Abstract

In this study, a high-Q circular substrate-integrated waveguide (SIW) cavity resonator is proposed as a non-contact and non-invasive radio frequency (RF) sensor for chemical sensing applications. The design of the structure utilizes SIW technology along with a circular shape to achieve a high unloaded Q factor, which is one of the important requirements for RF sensors. The resonant frequency of the proposed circular SIW cavity sensor changes when a liquid material or a chemical (microliters) is inserted in the sensitive area of the structure. The sensing of liquid materials with different permittivities is accomplished via the perturbation of the electric fields in the SIW configuration. When a microwell that is 4 mm in radius is installed vertically through the center of the bare circular SIW cavity, the operating frequency varies from 5.26 to 5.34 GHz. Similarly, when the microwell contains ethanol, the frequency shifts from 5.26 to 5.18 GHz, and the amplitude of reflection coefficient is shifted from −29 dB to −17 dB; when the microwell contains mixing deionized (DI)-water, the frequency moves from 5.26 to 4.98 GHz (which is also 0% Ethanol in our study), and the amplitude of reflection coefficient is shifted from −29 dB to −8 dB. A high unloaded Q factor is maintained throughout all experimental results. To demonstrate our idea, different concentrations of ethanol are tested and recorded. The experimental validation yields a close agreement between the simulations and the measurements.

## 1. Introduction

Chemical sensors have been used for many years to identify the purity ranks of numerous fluids to simplify the arrangement of those fluids for a wide range of industrial applications. It is necessary to store and classify these fluids or chemicals in accordance with the Globally Harmonized System of Classification and Labelling of Chemicals. The use of unidentified and unlabeled chemicals in experiments may have unforeseen consequences, and some of these chemicals may have severe effects on the human body. For example, methyl alcohol is toxic to the skin and body and can cause blindness, unconsciousness, and even death [1]. Consequently, fluidic chemical materials should be labeled so that they are recognized properly. Furthermore, a material safety manual should always be provided to the experimenters. 

Traditional liquid chemical sensors for investigating bioassays or chemical assays and determining liquid quality require a large amount of fluid for filling the taps or tubes [2,3]. Therefore, during the process of analysis and measurement, a large amount of fluid is wasted. To solve this problem of wastage, microfluidic structures have been proposed. A silicon-microfabricated diffusion-based photosensitive chemical device was proposed for measuring chemical concentrations [4]. This optical sensing device can measure the analytical concentrations in a tiny volume of a compound taster. Furthermore, a liquid-core optical ring-resonator device is discussed in [5]. A bonded silica tube acts as a ring resonator and transmits the fluid sample. The photosensitive characterization of the ring resonator is verified with a water–ethanol combination. This optically resonating device exploits the low liquid consumption, high sensitivity, and compact dimensions.

**Figure 1:**
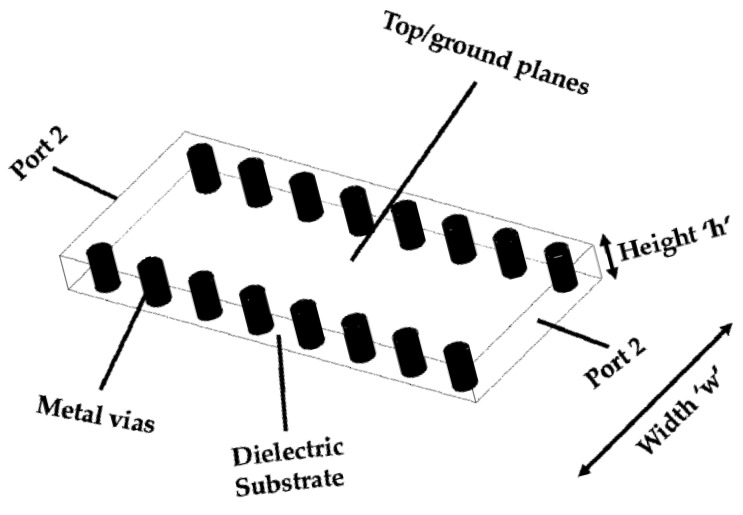
Illustration of the SIW structure.

Figure 1 presents the substrate-integrated waveguide (SIW) configuration. An SIW is a favorable contender for improvements in planar radiofrequency (RF) circuits for wireless communication applications [6,7,8,9,10,11,12]. Figure 1 clearly shows that the SIW structure comprises two walls of metallic vias that connect the bottom and top conductors, and there is very low-loss dielectric material between the two conductors [13]. Because of the simple planar fabrication and its easy integration with other circuits, SIW modules are widely preferred for low-loss and high-quality reflections, in addition to a metallic waveguide and planar printed circuit board (PCB). Compared with microstrip structures and co-planar striplines, SIWs are easy to handle, low-cost, easy to fabricate, easily miniaturized, and low-profile. SIWs also provide the advantages of the traditional metallic waveguide structure, i.e., a high quality factor (QF), lowermost losses, appropriate shielding, and all-out power-handling capability. One of the most important features of these structures is that a full circuit can be constructed in a planar configuration, which is very important for integration with other circuits, antennas, transitions, and rectangular or circular waveguides having planar configurations obtained through typical PCB fabrication procedures.

SIW modules also permit multiple mount-in chipsets on a single board, which helps them to be merged with diverse circuit systems that have dissimilar principles and reduce the total loss. Recently, excellent studies have been reported in the scientific community regarding the miniaturization of SIW cavities [14]. This extensive investigation has forced the enhancement of design methods in SIW technology, and a new technique and procedure were presented recently to make SIWs reusable and more compact [15,16,17,18].

Polydimethylsiloxane (PDMS) microfluidic reservoirs make it possible to perform liquid testing experiments on the microliter or nanoliter scale in RF electronics. A shift in the resonant frequency may be observed by just testing a very small quantity of the fluid. PDMS, which is a commercial silicone elastomer with a low Young’s modulus (<2 MPa), is a suitable candidate for making fluidic geometries [19]. PDMS exhibits a low surface energy and low modulus and is flexible. These properties allow it to be effortlessly attached onto electronic planes. It has a high loss tangent (0.0373) and a low dielectric constant (2.66) [20]. As previously mentioned, RF circuits permit the incorporation of fluidic reservoirs in order to sense different liquid materials, e.g., chemicals or bio-cells. An RF sensing device using E-shaped fluidic reservoir with a compact size and low loss was proposed in [21]. 

A very high-Q, non-invasive, and non-contact circular SIW cavity resonator is proposed for liquid chemical detection. The original configuration employs a circular waveguide combined with an SIW construction, which offers various features: (1) lightness; (2) low radiation losses; and (3) a high QF, as previously discussed [13]. A circular SIW was chosen during the parametric analysis, because it produced a high unloaded Q factor of 1080, which the other configurations (square, rectangular, and triangular) failed to produce. The circular shape is derived from the circular waveguide resonator theory [22]. By combining the circular waveguide resonator with SIW technology, a very high unloaded Q factor of 1080 was realized in a planar configuration. In the presented research work, a PDMS microwell is installed in the center of the circularly constructed SIW to achieve a higher frequency-shifting ability while maintaining a high Q. A PDMS microwell can accommodate a maximum of 3 µL of liquid material. Therefore, the operating frequency of the proposed device is significantly affected by testing a very small quantity of fluid at the center of the circular SIW cavity. Quality control is one of the commercial application for our proposed sensor [23], but it can also be utilized in local chemistry laboratories for identification and labeling of liquid chemicals. Microwave cavity modules with such a configuration have been previously reported, along with frequency-tunable RF resonators [24,25,26,27,28].

## 2. Sensor Design 

### 2.1. Circular SIW Cavity Resonator Design

The transverse magnetic (*TM*_01_) and transverse electric (*TE*_11_) modes are the dominant modes in a circular waveguide, as described in [29]. After deriving the Bessel’s differential equation and Bessel functions, we express the lowest cutoff frequency of the two dominant modes *TM*_01_ and *TE*_11_ as follows:

(1) $${\left(f_{c}\right)}_{TM_{01}}=\frac{0.383}{a\sqrt{\mu \varepsilon }}$$

(2) $${\left(f_{c}\right)}_{TE_{11}}=\frac{0.293}{a\sqrt{\mu \varepsilon }}$$

where *a* is the radius of the circular waveguide, and *μ* and *ε* are the permeability and permittivity, respectively, of the dielectric material. Simultaneously, the resonant frequency of dominant *TM*_nmℓ_ and *TE*_nmℓ_ modes in the circular waveguide cavity are defined as [29]:

(3) $$f_{nm\ell }=\frac{c}{2\pi \sqrt{\mu_{r}\varepsilon_{r}}}\sqrt{{\left(\frac{P_{nm}}{a}\right)}^{2}+{\left(\frac{\ell \pi }{d}\right)}^{2}}$$

(4) $$f_{nm\ell }=\frac{c}{2\pi \sqrt{\mu_{r}\varepsilon_{r}}}\sqrt{{\left(\frac{P\prime_{nm}}{a}\right)}^{2}+{\left(\frac{\ell \pi }{d}\right)}^{2}}$$

where *c* is the speed of light, *P*_*nm*_ and *P*’_*nm*_ are the roots of Bessel’s function *J*_n_(*x*) and *J*’_n_(*x*), *a* is the radius of the circular cavity, *d* is the depth, and *μ_r_* and *ε_r_* are the permeability and permittivity, respectively.

**Figure 2:**
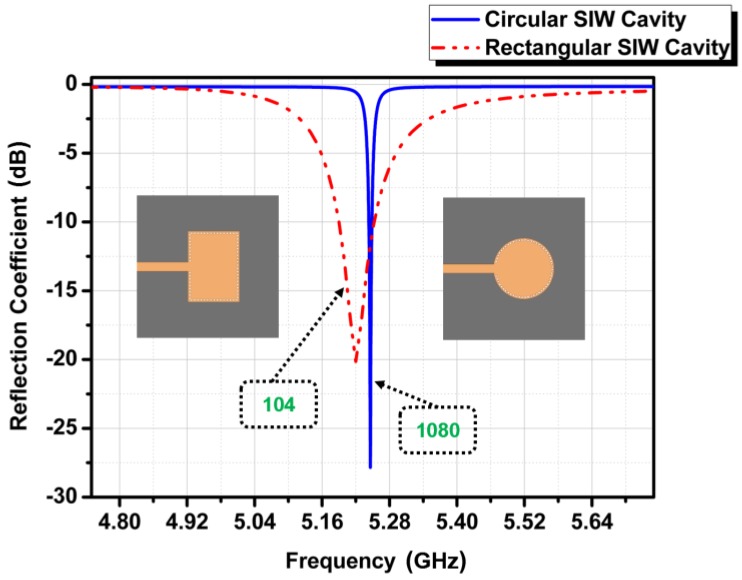
QF comparison between a rectangular SIW cavity and a circular SIW cavity at the same frequency.

Our proposed structure is a combination of a circular waveguide and SIW technology. The reason for not choosing a typical rectangular SIW cavity structure is its lower Q factor compared to that of the circular SIW cavity. Therefore, to achieve our goals of attaining higher unloaded Q factor and better ethanol sensitivity with SIW technology compared to RF resonators, a circular waveguide cavity resonator is used along with the SIW technology; the structure becomes planar, and thus has a *TE*_10_ as the dominant mode of operation in our proposed research. The QF comparison between rectangular and circular SIW cavities is shown in Figure 2.

The unloaded quality factor (Q) of a resonator can be defined as [29]:

(5) $$Q=2\pi f_{0}\frac{W_{m}+W_{e}}{P_{loss}}$$

where *f*_0_ is the resonant frequency and *P_loss_* is the dissipated power. *W_m_* and *W_e_* are the average stored magnetic and electric energies, respectively. The Q factor can be calculated from the 3 dB bandwidth as:

(6) $$Q=\frac{f_{0}}{f_{H,3dB}-f_{L,3dB}}$$

where *f*_H,3dB_ and *f*_L,3dB_ are the high and low frequencies at 3 dB below peak, respectively. In this work, we have used Equation (6) to calculate the unloaded Q factors for the simulated and measured reflection coefficient results. The above concept of unloaded Q-factor calculations for a one-port reflection resonator (our case) or for a two-port transmission and absorption cases is clearly explained in the these excellent works [30,31,32,33]. 

**Figure 3:**
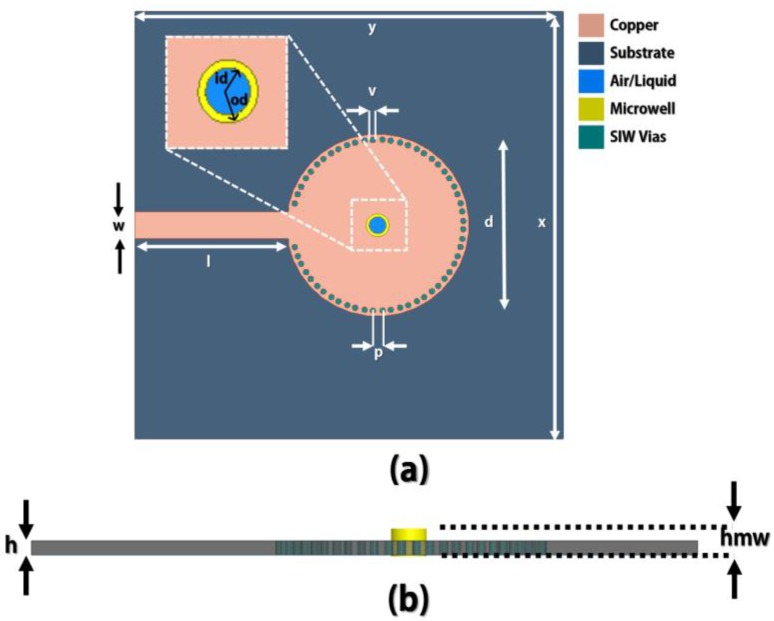
Design layout of the proposed sensor: (**a**) top view (zoomed microwell) and (**b**) cross-sectional view.

**Figure 4:**
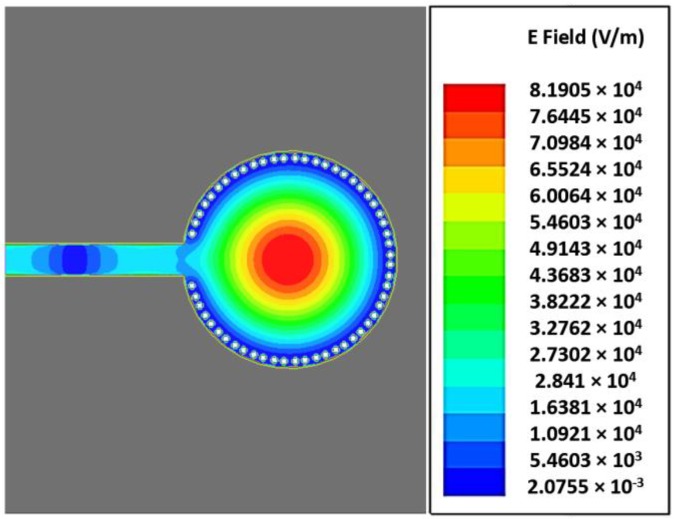
E-field distribution of the circular SIW resonator without microwell installed.

Figure 3 shows the design of the circular SIW cavity resonator. It is fed by a 50-Ω microstrip line. The Rogers RT/Duroid 5880 (Rogers Corporation, Connecticut, CT, USA) dielectric material (thickness = 1.575 mm, dielectric constant = 2.2, loss tangent = 0.0014) is selected to realize the idea. Initially, the structure was designed to resonate at 5 GHz. The detailed geometric parameters of the design are presented in Table 1. Figure 4 illustrates the electric-field (e-field) circulation of the simulated design when there is no microwell installed on the structure. Figure 4 clearly indicates that the magnitude of the e-field is concentrated at the center of the circular waveguide. A circular SIW cavity resonator is used in this study because of its lower scrounging loss, higher QF, and ease of PCB fabrication compared with other SIW shapes [14,17,34,35]. 

The operating frequency of the proposed device is directly proportional to the effective dielectric constant of the dielectric material. Furthermore, its effective permittivity is changed by testing fluidic chemicals or liquid biomaterials inside the microwell on the SIW cavity. Consequently, the suggested SIW cavity sensor can sense the permittivity of several fluidic chemicals by observing the change in the resonant frequency of the device. The e-fields of the structure are disturbed, which causes shifts in the frequency.

### 2.2. PDMS Microwell Design

**Figure 5:**
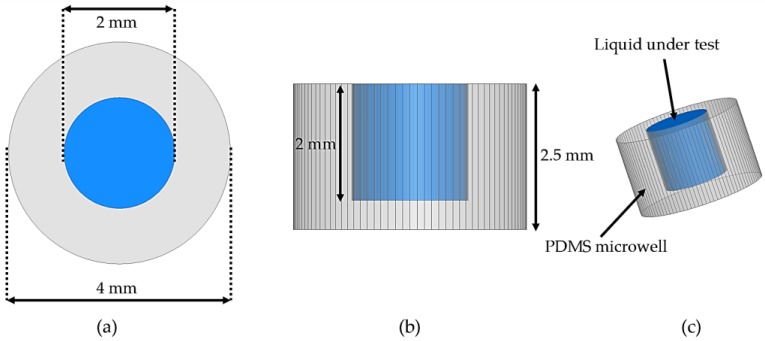
PDMS microwell: (**a**) top view, (**b**) cross-sectional view, and (**c**) bird’s-eye view.

A microwell with PDMS material is constructed and installed at a location where the magnitude of the e-field is strong (center). It is constructed to contain a maximum liquid volume of 3 µL. Each of the liquid samples tested in this study is 3 µL in volume. The microwell reservoir is 2 mm in height and 2 mm in diameter. As shown in Table 1, the diameter of the microwell is chosen to be 4 mm. This value is selected on the basis of parametric simulations, for checking the frequency sensitivity and maintaining a high Q, which are important features of an RF sensor. Figure 5 shows the detailed PDMS microwell construction for testing liquid materials.

**Figure 6:**
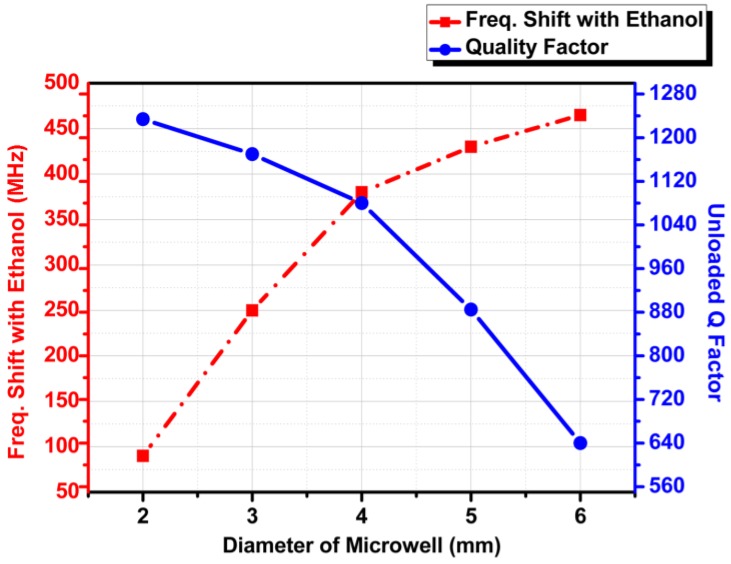
Simulated frequency shift and unloaded Q Factor at different diameters of the PDMS microwell.

To justify the dimensions of the microwell, the microwell diameter is chosen as 4 mm on the basis of a parametric study. Figure 6 shows the simulation results for the case where the outer diameter of microwell varied from 2 to 6 mm. It must be noted that outer diameter is always a double of inner diameter. For instance, when the outer diameter is 2 mm, the inner diameter is 1mm; and when the outer diameter is 3 mm, the inner diameter is 1.5 mm, respectively. The parametric study revealed that 4 mm was the optimal outer diameter (2 mm inner diameter as seen in Figure 6) for installing the microwell in the structure to maintain a suitable QF of the circular SIW cavity and allow a significant frequency shift (between ethanol filled and empty microwell).

## 3. Simulation Results

After the design process, a full wave computer simulation is performed using an ANSYS high-frequency structure simulator (HFSS). A microwell using a PDMS reservoir is examined, and its material specifications are extracted to produce a physical object in the computer software. Ethanol is also examined at a frequency of 5 GHz and is stored inside the microwell to validate our notion of sensing. The simulated reflection coefficients of the bare resonator (without the microwell), the ethanol-filled microwell, and the empty microwell are predicted to have distinct frequency responses.

**Figure 7:**
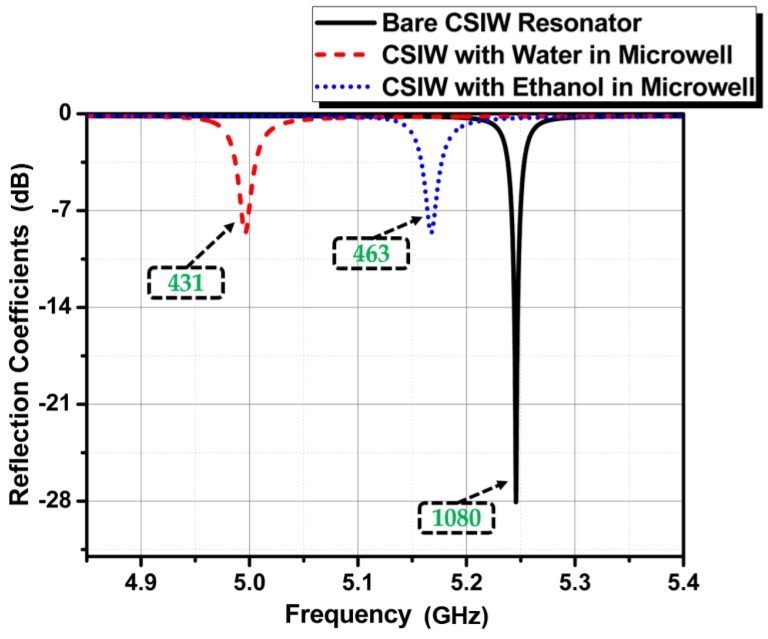
Simulated reflection coefficient for the bare circular SIW resonator, the water-filled microwell, and the microwell filled with ethanol. The unloaded Q factor is shown in a box for each result.

Figure 7 shows the frequency responses for the three aforementioned cases, where the circular SIW resonator does not have a microwell, has a water-filled microwell, and has a microwell reservoir filled with ethanol. The corresponding operating frequencies observed are 5.24, 4.99, and 5.16 GHz. The unloaded QFs for these three cases are 1080 (bare), 431 (water-filled), and 463 (ethanol), respectively. The structure exhibits a very high Q, which is important for the ability of RF sensors to detect liquid materials having a high permittivity. Consequently, it can be predicted that this sensor device can function as a chemical concentration sensor and can sense many liquid materials with dissimilar relative permittivity.

## 4. Experimental Demonstration

**Figure 8:**
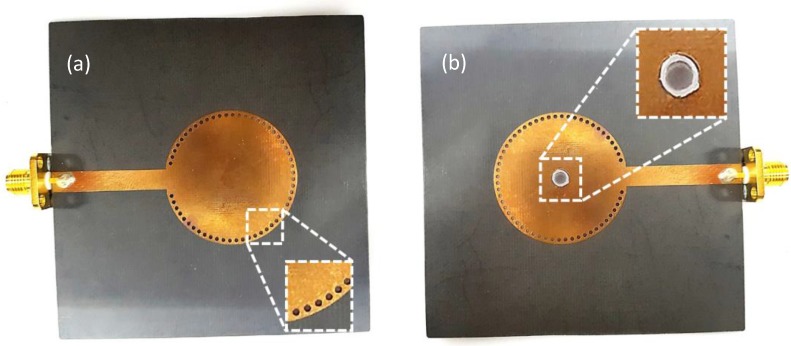
Fabricated sensor prototype: (**a**) circular SIW cavity resonator with metallic vias zoomed, and (**b**) with PDMS microwell in the center (microwell zoomed).

Figure 8 shows photographs of the proposed sensor. Figure 8a shows a fabricated circular SIW cavity resonator that has undergone a PCB etching process on a Rogers Duroid 5880 substrate. Figure 8b shows that a PDMS microwell has been inserted into the 4-mm hole at the center of the cavity. The drilled holes around the cavity are copper-plated metallic vias that connect the top copper patch with the bottom ground, making it an SIW cavity. 

**Figure 9:**
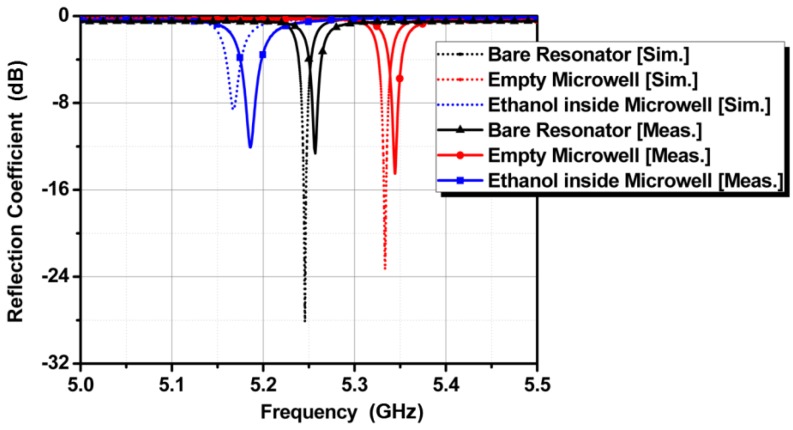
Measurement results plotted alongside simulated results.

Ethanol is injected in the microwell, and the frequency responses for the previously discussed three cases are logged using an HP 8510C vector network analyzer (VNA) manufactured by Hewlett Packard, Palo Alto, CA, USA. In Figure 9, the simulation results are compared with the measurement results. The measured resonant frequencies exhibit good agreement with the simulated resonant frequencies. We can observe the difference between the simulated and measured reflection coefficients due to error in imaginary values of complex permittivity. In general, the real values of complex permittivity determines the resonant frequency, while the imaginary values of complex permittivity determines the reflection coefficient. However, it is difficult to extract the accurate imaginary values of complex permittivity rather than the real values of complex permittivity.

**Figure 10:**
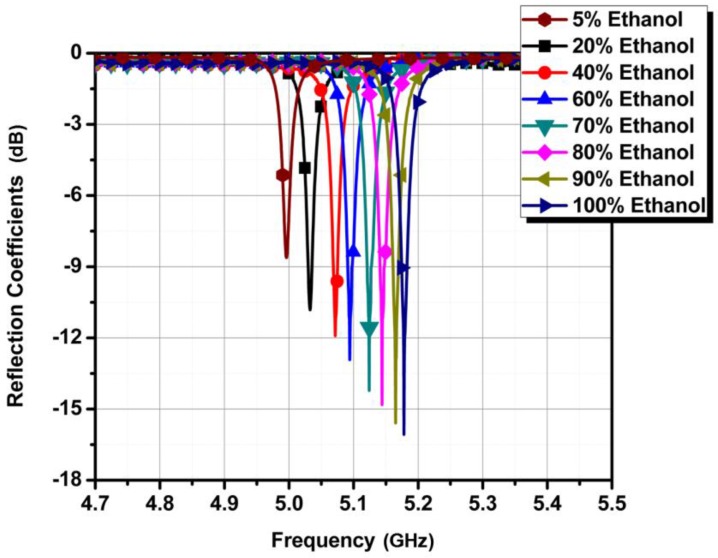
Measured reflection coefficients of different concentrations of ethanol, ranging from 5% to 100%.

Next, we aimed to employ this device as a purity sensor for ethanol. We prepared different concentrations of ethanol by mixing deionized (DI) water and measured the frequency responses. Figure 10 shows the measured reflection coefficients when the concentration of ethanol is sensed from 5% to 100%. The total amount of liquid that can fill the microwell is 3 µL, and 50% ethanol means 1.5 µL of ethanol mixed with 1.5 µL of DI water. 

**Figure 11:**
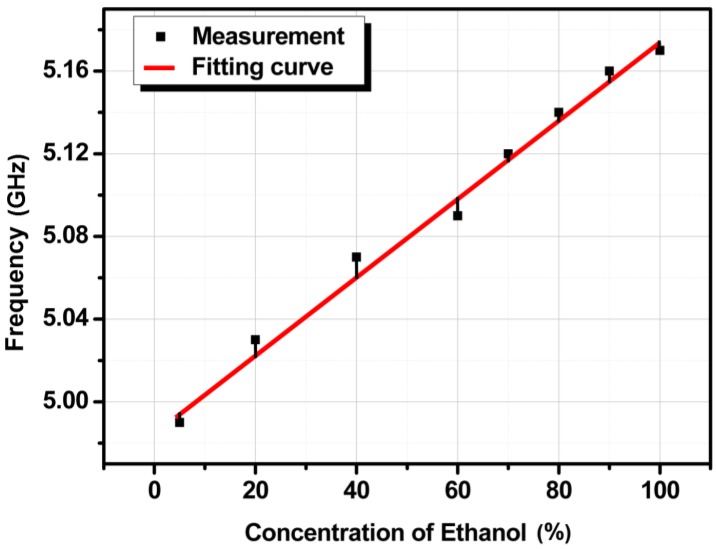
Frequency of the device with ethanol concentrations ranging from 5% to 100% with a fitting curve of *y* = 1.9 × 10^−3^ *x* + 4.98.

The lowest bound of chemical recognition for the proposed sensor is 5% ethanol. To prepare 5% ethanol, 1 mL of DI water is stirred with 50 µL of pure ethyl alcohol. Absolute grade ethanol solvent (CH_3_CH_2_OH, part number 32,205) was purchased from Sigma–Aldrich (St. Louis, MO, USA). This solvent contained 789 g of solute in 1 L of water [36], which means that 5% ethanol corresponds to 39,450 ppm in our experimental demonstration. The material loss of 100% ethanol is higher than the material loss of 5% ethanol; however, because of the very high Q of the proposed SIW resonator, the QF is decent and does not degrade with different chemical concentrations. The relationship between the frequency and the ethanol concentration is almost linear, described as *y* = 1.9 × 10^−3^ *x* + 4.98 for ethanol purities ranging from 5% to 100%. When the sensitivity of the proposed device as an ethanol sensor is resolute by the slope angle of the fitting curve, it is 1.9 × 10^6^ Hz/percentage. The sensitivity plot is shown in Figure 11 where error range is between −10 MHz to 10 MHz.

**Figure 12:**
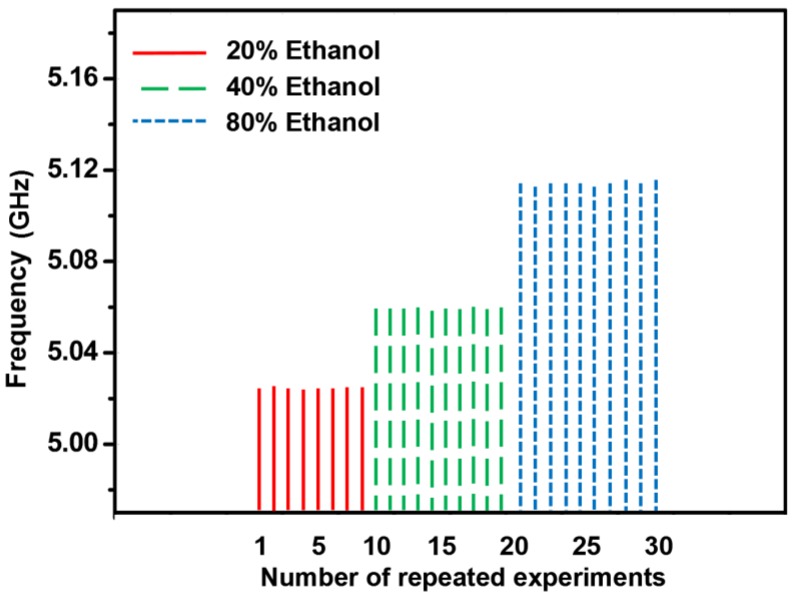
Repeatability test results of the fabricated sensor.

To demonstrate the repeatability of the sensor, 10 experiments for 20% ethanol, 40% ethanol, and 80% ethanol are repeatedly performed under the same measurement setup. For the three aforementioned ethanol concentrations, the resonant frequency remained the same after emptying the microwell and re-injecting the solution in the microwell. The room temperature was controlled to 22 °C throughout our experiments. Figure 12 shows that the proposed sensor is reliable and the measurement results are reproducible using the same fabricated prototype.

A comparison of proposed device with other recently reported SIW ethanol sensors is presented in Table 2. The unloaded Q factors are compared, when the resonators do not have an ethanol sample. All of the measurement results indicate that the proposed sensor device has a higher unloaded Q factor when there is no ethanol sample inside the resonator’s sensitive area (bare resonator). Our proposed circular SIW cavity sensor also achieved a largest frequency shift and highest sensitivity for ethanol chemical. Some highly appreciated works using GHz-THz frequency band, sensing biomaterials, petrol, propanol, and other aqueous solutions using both optical and RF sensing mechanism are mentioned here [38,39,40,41,42,43,44,45]. 

## 5. Conclusions

A chemical and chemical-purity sensor is proposed. The device employs a circular SIW configuration. A microfluidic microwell made of PDMS is installed at the most subtle location of the SIW cavity (center). When a microfluid (maximum of 3 µL) is dropped on the PDMS microwell, the frequency of the device changes because of the change in the effective dielectric constant. The proposed sensor is constructed on a Rogers/Duroid 5880 dielectric material using the typical PCB etching procedure, and the microwell is constructed over the PDMS material using a laser-etching engine, which is a simple and brief procedure. Our proposed idea is confirmed by close agreement between the simulation and measurement results. In addition, the measured results indicate a distinguishable frequency response when the ethanol purity is varied from 5% to 100% or when a different bio-cell is measured. The proposed sensor is planar, non-invasive, very high-Q, contracted, reusable, and non-contacting, and it accepts a very minute liquid sample for sensing. It is a low-cost device that can easily be manufactured through a typical PCB procedure. In this study, it was successfully demonstrated that the proposed circular SIW cavity sensor can detect chemicals and the purity level of ethanol. The repeatability and reliability of the presented sensor was confirmed by conducting more than 10 similar experiments. The proposed sensor discriminates various liquid chemicals having different dielectric constants. However, it is the limitation of RF sensors that they cannot differentiate between mixtures of chemicals or biomaterials having similar dielectric constants. Thus, we have proposed this device particularly as an ethanol concentration sensor. Quality control is one of the commercial applications for our proposed sensor, but it can also be utilized in local chemistry laboratories for identification and labeling of liquid chemicals. We are confident that in the future, we will produce this sensor on a flexible or inkjet-printed low-cost paper substrate for its use in wearable electronics or e-health because of increased advances. 

## Figures and Tables

**Table 1:** Detailed geometric parameters of the design.

Number	Parameter	Dimension (mm)	Description
1	x	75	Width of substrate and metallic bottom ground
2	y	75	Height of substrate and metallic bottom ground
3	l	27	Length of microstrip line feeding
4	w	4.75	Width of microstrip line feeding
5	d	32	Diameter of circular SIW patch
6	p	1.7	Pitch (center to center) distance between vias
7	v	0.45	Diameter of SIW vias
8	id	2	Inner diameter of microwell
9	od	4	Outer diameter of microwell
10	h	1.575	Height of Rogers substrate
11	hmw	2.5	Height of microwell

**Table 2:** Performance of the proposed sensor compared with other SIW-based ethanol sensors.

Reference	Sensing	Frequency Shift (∆*f*) ^ǂ^ (MHz)	Sensitivity (MHz/*ε_r_*)	Technology	QF	Volume (μL)
[21]	Ethanol liquid	145	26.36	SIW	39.12	10
[34]	Ethanol liquid	70	12.73	SIW	51	1
[37]	Ethanol liquid	38	5.84	SIW	334.6	500,000
This work	Ethanol purity	380	69.07	Circular SIW	1080	3

^ǂ^ Frequency shift (∆*f*) and sensitivity (∆*f*/∆*ε_r_*) are calculated from air and ethanol; *ε_r_* of air and ethanol are 1 and 6.5, respectively [38]. Unloaded QF are calculated when there is no ethanol sample (bare resonator).
